# The Role of Stress Management in the Relationship between Purpose in Life and Self-Rated Health in Teachers: A Mediation Analysis

**DOI:** 10.3390/ijerph13070719

**Published:** 2016-07-16

**Authors:** Fei Li, Jieyu Chen, Lin Yu, Yuan Jing, Pingping Jiang, Xiuqiong Fu, Shengwei Wu, Xiaomin Sun, Ren Luo, Hiuyee Kwan, Xiaoshan Zhao, Yanyan Liu

**Affiliations:** 1 School of Traditional Chinese Medicine, Southern Medical University, Guangzhou 510515, Guangdong, China; leephialf@126.com (F.L.); jieyu@smu.edu.cn (J.C.); niuniu_jing@126.com (Y.J.); 13427550499@163.com (P.J.); a2278197a@163.com (S.W.); sunxiaomin198001@163.com (X.S.); luoren2014@126.com (R.L.); 2 Department of Traditional Chinese Medicine, The Affiliated Brain Hospital of Guangzhou Medical University (Guangzhou Huiai Hospital), Guangzhou 510170, Guangdong, China; yulinfimmu@126.com; 3 School of Chinese Medicine, Hong Kong Baptist University, Hong Kong 999077, China; 13480405@life.hkbu.edu.hk (X.F.); hykwan@hkbu.edu.hk (H.K.)

**Keywords:** purpose in life, stress management, self-rated health

## Abstract

*Background*: To examine whether stress management mediates the relationship between purpose in life and self-rated health status (SRH). *Methods*: A cross-sectional survey was conducted among 6840 teachers in 2013 in Guangzhou, China. Purpose in life was assessed through the Purpose in Life Subscale of the Psychological Well-being Scale. Stress management was assessed using the eight-item questionnaire adapted from the Health-promoting Lifestyle Profile II. SRH was assessed by the Suboptimal Health Measurement Scale Version 1.0. The mediation hypothesis was tested by the structural equation model for path analysis. *Results*: It was found that purpose in life had direct and indirect effects on SRH. The path analysis showed the total effect (β = 0.563) of purpose in life on SRH was comprised of a direct effect (β = 0.319) and an indirect effect (β = 0.244), which was mediated by stress management. *Conclusions*: By supporting the mediation hypothesis, our results indicate that stress management mediated the effect of purpose in life on SRH. Enhancement of teachers’ purpose in life and improvement of training skills of stress management should be incorporated in the strategy of improving teachers’ health.

## 1. Introduction

Purpose in life is a self-organizing life aim that stimulates goals, manages behaviors, and provides a sense of meaning [1]. Studies have found that higher purpose in life is associated with better health outcomes and better self-rated health (SRH) [2]. SRH is a self-assessed or self-perceived health status, providing a good reflection of “subjective” or “perceived” health [3], which focuses on the evaluation of a group’s health status and an individual’s well-being. Individuals with high purpose in life strongly feel more in charge of their health [4,5,6] and believe in the ability to influence their health [7]. Physiologically, plenty of evidence has shown that individuals with high purpose in life may live longer, and have a reduced the risk of debilitating conditions [8,9,10] and decreased mortality [11]. Psychologically, a growing body of studies indicates that purpose in life has strong associations with psychological well-being and a low level of depressive symptoms [12,13,14]. Socially, purpose in life was also reported as positively associated with social integration and relational quality [15].

Studies have shown that individuals with higher levels of education had higher purpose in life [16]. Teachers, as engineers of the human soul, not only have high education but also have the duty to cultivate a new generation and they also contribute to the stability of society. Therefore, teachers also have high purpose in life and high expectations in their work performance, which are reflected by students’ exam scores and the number of students admitted to prestigious schools. To achieve their purpose and expectation, they always suffer from heavy responsibility, excessive workload, time pressures and high expectations from both society and the students’ parents. Compared to other occupations, teachers were reported to have a higher level of stress [17]. Occupational stress induces worsening psychological conditions for teachers, and they were reported to have a higher prevalence of psychological distress [18]. Compared with other occupational populations, teachers in China reported the poorest SRH [19]. Hence, it is important for administrators, clinicians and researchers to note that improving teachers’ skills of stress management seems to be crucial to enhance their health.

Stress management involves changing the stressful situation, dealing with problems, taking care of yourself, and making time for rest and relaxation. Stress management is reported to be positively associated with better SRH [20]. Studies suggest that stress management is related to improved immune function [21]. Good stress management capability is also reported to be associated with lower body mass index and restful sleep in the elderly [22], improving chronic neck pain [23], reducing healthcare utilization [24], leading to a higher level of psychological well-being [25,26,27] and increased social support from others [28]. Individuals with better stress management can promote their health.

Therefore, our hypothesis is that stress management could be a potential explanatory factor in the association between purpose in life and SRH. The current study aimed to investigate the relationship between purpose in life, stress management and SRH and the effect of stress management on the relationship between purpose in life and SRH in teachers.

## 2. Subjects and Methods

### 2.1. Subjects and Data Collection

A cross-sectional, descriptive survey was conducted among 8111 public school teachers during April to July 2013 in Guangzhou, the capital of Guangdong Province, South China. Three districts (i.e., Luogang, Liwan and Zengcheng) were randomly selected from 12 in Guangzhou and are representative of economic characteristics, population demographics, and geographic distribution. Then all the primary and secondary public schools in each area were chosen. An additional 1271 participants were removed due to inconsistent or potentially biased responding, and missing or incomplete information. Finally, 6840 teachers were included in the current analysis, resulting in a valid response rate of 84.3%.

### 2.2. Measurement of SRH

SRH was assessed via the Suboptimal Health Measurement Scale Version 1.0 (SHMS V1.0), which was developed by our research group. According to Chinese research data, the SHMS V1.0 has displayed an excellent level of content reliability and validity, supported by Cronbach’s alpha and split-half reliability coefficients of 0.917 and 0.831, respectively [29]. These statistics indicate that SHMS has good reliability. The scale comprises 39 items, pertaining to three subscales: (1) physiological symptoms (14 items); (2) psychological symptoms (12 items); and (3) social symptoms (nine items), with four items for subjective health status, whereby participants are asked: “What is your general feeling in terms of physiological/psychological/social/general health?” Items are rated on a five-point Likert scale (i.e., “none”, “occasionally”, “sometimes”, “constantly” and “always”), with each item scored from 1 to 5. Transformed scores are used to account for reverse questions. Higher scores represent better SRH status. The overall scale of Cronbach’s alpha in this study was 0.935 with subscale alphas of self-rated physiological (α = 0.854), psychological (α = 0.891), and social (α = 0.872) health.

### 2.3. Measurement of Purpose in Life

Purpose in life was assessed through the Purpose in Life Subscale of the Psychological Well-being Scale [30,31], with high internal consistency for the six domains (Cronbach’s alpha’s from 0.86–0.93) and good test-retest reliability with Pearson product moment coefficients over a six-week period ranging from 0.81–0.88. The subscale consisted of three items: “Some people wander aimlessly through life, but I am not one of them”; “I live life one day at a time and I don’t really think about the future (reversed)”; and “I sometimes feel as if I’ve done all there is to do in life (reversed)”. The response option was modified as a four-point variable (from “strongly disagree” to “strongly agree”. The total purpose in life scores ranged between 3 and 12.

### 2.4. Measurement of Stress Management

Stress management was measured through an eight-item questionnaire (e.g., “I use some skills to deal with my stress”), adapted from the Health-Promoting Lifestyle Profile II, which was developed by Walker [32]. The response was on a four-point Likert scale from 1 (never) to 4 (routinely). Total scores are calculated; higher scores indicate better stress management. In the current study, the Cronbach’s alpha value was 0.808, comparing favorably with previous reported alpha values 0.75 [33].

### 2.5. Statistical Analysis

Descriptive statistics including means, standard deviations, and frequencies were calculated. Bivariate correlations were calculated using Pearson correlation coefficients to examine relationships among purpose in life, stress management, SRH and dimensions of SRH. The structural equation model (SEM) for path analysis was constructed to analyze the direct and indirect effects of purpose in life on SRH. A model was established with purpose in life as the independent variable, SRH as the dependent variable and stress management as the mediating variable. Confirmatory factor analysis (CFA) was run on the subscales. All significance tests were two-sided, with *p*-values < 0.05 considered statistically significant. The analyses were conducted using SPSS 13.0 (SPSS Inc., Chicago, IL, USA) and AMOS 22.0 (SPSS Inc., Chicago, IL, USA) software.

### 2.6. Ethic Statement

The study was approved by the ethics committee of Nanfang Hospital in Guangzhou, China (2012) LunShenZi (No. 035). It complied with the principles outlined in the Helsinki Declaration. All procedures were performed in accordance with the ethical standards. Written consent was obtained from each participant.

## 3. Results

### 3.1. Sample Characteristics and Correlations between Study Variables

Sample characteristics are shown in Table 1. Pearson’s correlation analysis of the purpose in life, stress management, SRH and dimensions of SRH scores are reported in Table 2. Purpose in life had a strong and positive correlation with stress management (r = 0.589, *p* < 0.001), while it had a moderate and positive correlation with SRH (r = 0.462, *p* < 0.001), and self-rated physiological (r = 0.302, *p* < 0.001), psychological (r = 0.448, *p* < 0.001) and social (r = 0.471, *p* < 0.001) health. Stress management showed a strong and positive correlation with SRH (r = 0.547, *p* < 0.001), self-rated psychological health (r = 0.511, *p* < 0.001), while it had a moderate and positive correlation with self-rated physiological (r = 0.416, *p* < 0.001) and social (r = 0.498, *p* < 0.001) health.

### 3.2. Confirmatory Factor Analysis of the Subscales

Table 3 shows the results of the confirmatory factor analysis of the subscales. The standardized estimate of each item was generally >0.4 (*p* < 0.001). These statistics indicate that the items can reflect the subscales. The composite reliability (CR) of purpose in life, stress management, self-rated physiological, psychological and social health were 0.798, 0.813, 0.840, 0.876 and 0.861, respectively. The CRs were all >0.7, which revealed that the subscales have good internal consistency reliability and validity.

### 3.3. Structural Equation Modeling

**Figure 1:**
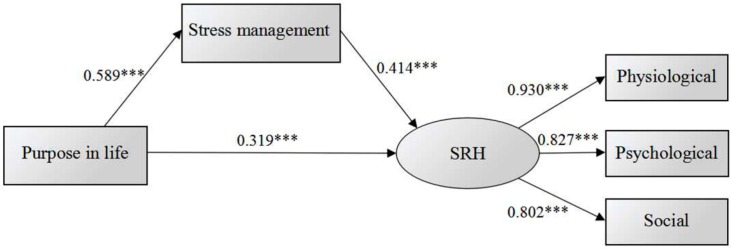
The structural equation model of the relationship between purpose in life, stress management and SRH. (*** *p* < 0.001).

The structural equation model was constructed and is shown in Figure 1. Table 4 provides path coefficients between various structural variables. Fit indices of the model are presented in Table 5, which revealed a good fit of the data with SRMR (standardized root mean square residual) < 0.08, GFI (goodness-of-fit index) > 0.90, AGFI (adjusted goodness-of-fit index) > 0.90, NFI (normed fit index) > 0.90, IFI (incremental fit index) > 0.90, TLI (Tucker-Lewis index) > 0.90, CFI (comparative fit index) > 0.90, and RMSEA (root mean square error of approximation) < 0.08. From Figure 1 and Table 4, purpose in life had a positive effect on SRH, which was mediated by stress management. The total effect (β = 0.563) of purpose in life on SRH was comprised of not only its direct effect (β = 0.319), but also the indirect effect (β = 0.244) generated by stress management. The standardized estimations of SRH on self-rated physiological (β = 0.930), psychological (β = 0.827) and social (β = 0.802) health were all >0.7, which indicated that self-rated physiological, psychological and social health can effectively reflect SRH. Therefore, stress management also partially mediated the effects of purpose in life on self-rated physiological, psychological and social health.

## 4. Discussion

The study aimed to investigate the relationship between purpose in life, stress management and SRH, and the effect of stress management on the relationship between purpose in life and SRH. In the current study, we found that purpose in life positively and directly affected SRH among teachers. Stress management also plays a partial mediator role in the relationship between purpose in life and SRH.

Firstly, purpose in life had a positive and direct effect on SRH in our study. Correlation analysis also indicated that teachers showed a moderate but significantly positive correlation between purpose in life and SRH. Previous studies have indicated that persons with high purpose in life report better SRH [2] and have better health outcomes (e.g., better immune function, reduced risk of debilitating conditions and mortality) [8,9,11,34]. Studies also indicate that purpose in life has a strong association with psychological well-being [13]. Schaefer et al. [12] suggested that greater purpose in life predicts a better recovery from negative stimuli. Similarly, Pietrzak and Cook [14] found that purpose in life may help to promote psychological resilience in older veterans who have endured a significant number of life traumas. Furthermore, purpose in life was also reported to positively affect self-rated social health. Individuals with high purpose in life have better social integration, relational quality, social participation and stronger social support [15,35]. These previous findings were supported by current findings.

Secondly, our data indicated that purpose in life affected SRH indirectly through the mediation of stress management. To our knowledge, this study is the first to indicate the mediating effect of stress management on the relationship between purpose in life and SRH. On one hand, our study found that stress management was significantly directly affected by purpose in life. It has been reported that individuals with higher purpose in life have better skills for dealing with stress and facilitating recovery from stress [36], have greater influence over the function of the autonomic nervous system, and can reduce the development of anxiety [37]. The results also supported these previous findings. On the other hand, results indicated that stress management also significantly directly affected SRH, which was in line with previous studies showing that individuals with better stress management behaviors reported better subjective health status [20]. McGregor et al. found that women with early-stage breast cancer had improved lymphocyte proliferation after a 10-week stress management intervention [21]. Good stress management capability was also reported to be associated with lower body mass index and restful sleep in the elderly [22]. Stress management was also found to have strong associations with psychological well-being by reducing anxiety and depression, enhancing psychological hardiness and self-efficacy, and improving the overall mental quality of life [26,27]. Furthermore, Shimazu et al. conducted a stress management program among teachers and the results showed that the stress management program can contribute to increasing social support from co-workers [28]. Therefore, stress management played a mediator role in the relationship between purpose in life and SRH. Individuals with higher purpose in life have better skills of stress management to blunt the negative impact of stress on both body and mind, potentially leading to better SRH.

The results of the structural equation modeling confirmed stress management as a partial mediator of the relationship between purpose in life and SRH in teachers. There could be several reasons for this partial mediation. First of all, purpose in life had a direct effect on SRH in the model. Additionally, researches show that people with higher purpose in life may engage in healthier behaviors (e.g., exercising more, having more physical examinations, participating in preventive health services and acquiring adequate relaxation) [5,6,38,39], which results in better health conditions. Furthermore, previous studies of purpose in life have been shown to be associated with psychological outcomes such as well-being, happiness and life satisfaction [31,40,41]. The effects of purpose in life on health are expansive and influence numerous aspects of health. Thus, the mediation effect of stress management was only partial.

Several limitations should be considered when interpreting results from the current study. First, this study was of cross-sectional design. A replication of the mediation effect in a longitudinal study is necessary to consolidate the hypothesized directions of the causal relationships within the mediation model. Second, as with any self-report questionnaires, the data obtained in the current study may contain information bias. Future studies should address these limitations.

## 5. Conclusions

In summary, our findings indicated that purpose in life had direct and indirect effects on SRH. The indirect effect of purpose in life on SRH was mediated by stress management. In regard to the role and importance of purpose in life and stress management, besides the enhancement of teachers’ purpose in life, the improvement of training skills of stress management should also be incorporated in the strategy of improving teachers’ health.

## Figures and Tables

**Table 1:** Sample characteristics (*n* = 6840).

Variable	Total (*n* = 6840)	
	*n*	%
Sex		
Male	2518	36.8
Female	4322	63.2
Marital status		
Single	552	8.1
Married	6288	91.9
Education level		
College degree or below	2093	30.6
Bachelor degree or above	4747	69.4
Age, mean (SD)	38.24 (7.46)	
Body mass index	22.45 (3.10)	
Purpose in life, mean (SD)	8.75 (2.17)	
Stress management, mean (SD)	20.29 (4.18)	
SHS, mean (SD)	65.42 (12.03)	
Self-rated physiological health, mean (SD)	66.97 (13.35)	
Self-rated psychological health, mean (SD)	63.98 (15.54)	
Self-rated social health, mean (SD)	64.93 (14.17)	

**Table 2:** Pearson’s correlation analysis between purpose in life, stress management, SRH and dimensions of SRH.

Variables	1	2	3	4	5	6
1. Purpose in life	-	0.589 ***	0.462 ***	0.302 ***	0.448 ***	0.471 ***
2. Stress management		-	0.547 ***	0.416 ***	0.511 ***	0.498 ***
3. SRH			-	0.866 ***	0.915 ***	0.780 ***
4. Self-rated physiological health				-	0.681 ***	0.463 ***
5. Self-rated psychological health					-	0.655 ***
6. Self-rated social health						-

*** *p* < 0.001.

**Table 3:** The confirmatory factor analysis of the subscales.

Items		Subscales	Estimate	S.E.	Standardized Estimate	C.R.	*p*	CR
1	<---	Purpose in life	1.087	0.021	0.777	50.996	<0.001	0.798
2	<---		0.94	0.019	0.713	50.048	<0.001	
3	<---		1		0.772			
4	<---	Stress management	0.481	0.019	0.33	25.106	<0.001	0.813
5	<---		0.74	0.017	0.567	42.775	<0.001	
6	<---		0.64	0.016	0.516	39.041	<0.001	
7	<---		0.671	0.019	0.46	34.891	<0.001	
8	<---		0.715	0.018	0.539	40.769	<0.001	
9	<---		1.031	0.018	0.773	57.062	<0.001	
10	<---		1.033	0.018	0.787	57.901	<0.001	
11	<---		1		0.718			
12	<---	Self-rated physiological health	0.677	0.025	0.402	26.658	<0.001	0.84
13	<---		0.985	0.031	0.503	31.536	<0.001	
14	<---		0.89	0.036	0.371	25.007	<0.001	
15	<---		0.883	0.027	0.531	32.661	<0.001	
16	<---		0.966	0.029	0.542	32.933	<0.001	
17	<---		0.965	0.029	0.534	32.785	<0.001	
18	<---		1.191	0.032	0.669	37.786	<0.001	
19	<---		1.235	0.034	0.636	36.743	<0.001	
20	<---		1.116	0.032	0.57	34.387	<0.001	
21	<---		0.932	0.029	0.511	31.868	<0.001	
22	<---		0.964	0.03	0.528	32.589	<0.001	
23	<---		0.873	0.029	0.478	30.371	<0.001	
24	<---		0.819	0.02	0.483	40.787	<0.001	
25	<---		1		0.537			
26	<---	Self-rated psychological health	1.692	0.05	0.611	33.823	<0.001	0.876
27	<---		1.319	0.042	0.527	31.069	<0.001	
28	<---		1.313	0.042	0.535	31.36	<0.001	
29	<---		1.565	0.048	0.577	32.766	<0.001	
30	<---		1.397	0.042	0.593	33.225	<0.001	
31	<---		1.414	0.04	0.683	35.764	<0.001	
32	<---		1.788	0.049	0.712	36.306	<0.001	
33	<---		1.69	0.047	0.709	36.224	<0.001	
34	<---		1.915	0.053	0.71	36.422	<0.001	
35	<---		1.714	0.048	0.68	35.724	<0.001	
36	<---		1.126	0.031	0.46	36.231	<0.001	
37	<---		1		0.483			
38	<---	Self-rated social health	1.325	0.041	0.638	32.143	<0.001	0.861
39	<---		1.483	0.045	0.693	33.283	<0.001	
40	<---		1.32	0.041	0.647	32.215	<0.001	
41	<---		1.344	0.042	0.65	32.354	<0.001	
42	<---		1.741	0.052	0.692	33.343	<0.001	
43	<---		1.564	0.049	0.636	32.027	<0.001	
44	<---		1.656	0.05	0.7	33.436	<0.001	
45	<---		1.322	0.036	0.617	37.057	<0.001	
46	<---		1		0.458			

**Table 4:** The path coefficients between structural variables.

Path			Estimate	S.E.	Standardized Estimate	C.R.	*p*
Stress management	<---	Purpose in life	1.132	0.019	0.589	60.241	<0.001
SRH	<---	Purpose in life	1.817	0.09	0.319	20.13	<0.001
SRH	<---	Stress management	1.227	0.038	0.414	32.17	<0.001

**Table 5:** Fit indices for the structural models.

^2^/df	SRMR	GFI	AGFI	PGFI	NFI	IFI	TLI	CFI	RMSEA
25.721	0.011	0.997	0.978	0.133	0.996	0.997	0.983	0.997	0.06

SRMR: standardized root mean square residual; GFI: goodness-of-fit index; AGFI: adjusted goodness-of-fit index; PGFI: parsimony goodness of fit index; NFI: normed fit index; IFI: incremental fit index; TLI: Tucker-Lewis index; CFI: comparative fit index; RMSEA: root mean square error of approximation.
